# The fine spatial and temporal control of ultrasonic neuromodulation is a necessary condition for discerning the accompanying subjective feelings, but is it sufficient?

**DOI:** 10.1186/s40349-018-0118-2

**Published:** 2018-11-23

**Authors:** Robert Muratore

**Affiliations:** Quantum Now LLC, Huntington, New York USA

*“...one man can give birth to the elements of an art, but only another can judge how they can benefit or harm those who will use them.” - Plato, Phaedrus 274e*

Engineering models of biological systems can lead to better understanding and manipulation of those systems. The mechatronics systems model closely parallels the human nervous system [1]; the electromechanical feedback loops can be used to model the many biological arcs. Anatomy and physiology texts typically divide the nervous system into receptors, the sensory division of the peripheral nervous system, the central nervous system, the somatic and autonomic subdivisions of the motor division of the peripheral nervous system, the effectors, and, implicitly, the outside world. Guided by the mechatronics viewpoint on this ur-system, external, engineered energies (such as those available from electric, magnetic, and acoustic fields) can be injected directly into any stage of the nervous system loop. Thus it is possible to stimulate receptors in novel ways [2, 3], or to directly stimulate an upstream stage, bypassing the sensory apparatus [4, 5].

Such an injection of energy into the nervous system is an encompassing definition of neuromodulation [6, 7]. At low doses, neuromodulation is safe enough for consumer use, with commercially available low voltage direct current devices [8]. Subjective perceptions of such neuromodulation are subtle and muted, perhaps even lost in the thermal noise of the chemical processes that constitute thought [9].

Ultrasound has certain advantages as a neuromodulation modality, with finer spatial and temporal control compared to electromagnetic approaches [10] (offset by disadvantages such as the need for a coupling medium, the calculations needed to account for field distortions from the skull transit [11], and FDA strictures [12]).

The millimetre and millisecond resolution of ultrasonic neuromodulation matches important brain structures and functions and therefore could elicit clearer subjective phenomena. How could these phenomena be described?

Stimulus to a sensory cortex region is likely to be interpreted as the sense associated with that area. For example, stimulus to the tonotopic area of auditory cortex [13] would presumably result in a hearing sensation. If so, a sufficiently focused and lively ultrasound beam could “play” a tune by ranging over the tonotopic region.

More generally, traditional subjective measures of sensory phenomena [14] can provide some guidance, as can rigorous psychophysical methods for estimating thresholds and just noticeable differences [15].

However, these approaches might be challenging for stimuli outside of sensory regions if there is no vocabulary for the subjects and no set of previous experiences with which to compare. Even when words and images are available, certain subjective experiences, such as dreams, can elude rational description. Synesthesia is a possibility, mapping the new sensations onto existing sensory interpretations, but is it also possible to develop a new sense?

The brain plasticity required for the development of such a sense would require some learning in order to build up the necessary connections [16]. The brain has been shown to reroute around damaged regions in the hearing centre, and there is evidence of plasticity in the adult brain due to external stimulus [17].

Insofar as neuromodulation represents an external source that influences the brain, it can be considered to be the source of new, artificial senses [18, 19]. Each of the natural senses has highly developed cultural associations [20], and each sense has been amplified by technology. Artists have been at the forefront, almost by definition of their calling, of the interaction of the sensory amplification technologies and the cultural implications [21].

So how then can the possible new sensations be discerned? How can they be defined, and connected together to form feedback loops and networks embodying ideas of interest and of value? This is a role that artists can play. If a safe, effective neuromodulation apparatus could be provided, collaboration between bioengineers and artists can help to define the new neuromodulated perceptions.
